# Synthesis and Evaluation of ^99m^Tc-Labeled Dimeric Folic Acid for FR-Targeting

**DOI:** 10.3390/molecules21060817

**Published:** 2016-06-22

**Authors:** Zhide Guo, Mengna Gao, Manli Song, Changrong Shi, Pu Zhang, Duo Xu, Linyi You, Rongqiang Zhuang, Xinhui Su, Ting Liu, Jin Du, Xianzhong Zhang

**Affiliations:** 1 Department of Isotopes, China Institute of Atomic Energy, P. O. Box 2108, Beijing 102413, China; guozhide0518@gmail.com; 2 Center for Molecular Imaging and Translational Medicine, State Key Laboratory of Molecular Vaccinology and Molecular Diagnostics, School of Public Health, Xiamen University, Xiang’an South Rd, Xiamen 361102, China; 18037005951@163.com (M.G.); songmanli199142@126.com (M.S.); changrongshi2016@yahoo.com (C.S.); 18811471208@126.com (P.Z.); 13164604466@163.com (D.X.); youly01@sina.com (L.Y.); zhuangrongqiang@126.com (R.Z.); tingliu20072008@yahoo.com (T.L.); 3 Department of Nuclear Medicine, Zhongshan Hospital Affiliated of Xiamen University, Hubin South Road, Xiamen 361004, China; suxinhuii@163.com

**Keywords:** folate receptor, molecular imaging, dimeric folic acids, click reaction, SPECT imaging

## Abstract

The folate receptor (FR) is overexpressed in a wide variety of human tumors. In our study, the multimeric concept was used to synthesize a dimeric folate derivative via a click reaction. The novel folate derivative (HYNIC-D_1_-FA_2_) was radiolabeled with ^99m^Tc using tricine and trisodium triphenylphosphine-3,3′,3″-trisulfonate (TPPTS) as coligands (^99m^Tc-HYNIC-D_1_-FA_2_) and its *in vitro* physicochemical properties, *ex vivo* biodistribution and *in vivo* micro-SPECT/CT imaging as a potential FR targeted agent were evaluated. It is a hydrophilic compound (log P = −2.52 ± 0.13) with high binding affinity (IC_50_ = 19.06 nM). Biodistribution in KB tumor-bearing mice showed that ^99m^Tc-HYNIC-D_1_-FA_2_ had high uptake in FR overexpressed tumor and kidney at all time-points, and both of them could obviously be inhibited when blocking with free FA in the blocking studies. From the *in vivo* micro-SPECT/CT imaging results, good tumor uptake of ^99m^Tc-HYNIC-D_1_-FA_2_ was observed in KB tumor-bearing mice and it could be blocked obviously. Based on the results, this new radiolabeled dimeric FA tracer might be a promising candidate for FR-targeting imaging with high affinity and selectivity.

## 1. Introduction

The realization of accurate early detection of tumors requires the use of efficient and safe molecular imaging probes. To further improve the delivery efficiency and cancer specificity, several tumor targeting ligands (such as antibodies, sugars, folic acid (FA), transferrin, epidermal growth factor, and RGD peptide) are currently being pursued. One of the rational ligands is FA, which is a stable, inexpensive, and non-immunogenic vitamin (Mw = 441 Da). This ligand provides high tumor binding affinity and better tumor uptake for its corresponding radiotracers because of the enrichment of the folate receptor (FR) on the surface of many many tumors (e.g., ovarian, breast, colorectal, endometrial and renal carcinomas) [1,2,3]. In the field of folate-based radiopharmaceuticals, over the years, several probes for positron emission tomography (PET) or single-photon emission computed tomography (SPECT) have attracted significant interest, and become important tools in molecular imaging [4,5,6]. Folate conjugates also display this property after chemical modification, which makes FA an ideal structure for nuclear imaging [7,8,9,10].

In a previous study, we reported a ^99m^Tc-labeled folate derivative (^99m^Tc-HYNIC-T-FA) with a 1,2,3-triazole linkage [11]. Azide-functionalized HYNIC was coupled with alkyne-functionalized folate via a click reaction to give the final HYNIC-T-FA compound, which was proved to be a highly efficient method. Furthermore, ^99m^Tc-HYNIC-T-FA showed favorable characteristics compared with other folate derivatives modified with a HYNIC-binding entity. Although good evaluation results were obtained both *in vivo* and *in vitro*, there was still some room for further improvement of targeting efficiency. Introducing the multimeric cyclic RGD peptides provides higher α*_v_*β_3_ binding affinity and better tumor uptake for their corresponding radiotracers [12,13,14,15]. Inspired by the multimerization concept of cyclic RGD peptides, it might be a good strategy to improve the binding affinity and tumor targeting efficacy of tracers. To the best of our knowledge, no multimeric FR-targeting probes have been reported before. Therefore it is of interest to develop multimeric folate derivatives for radiolabeling and potential clinical applications.

In this study, a new FR-targeting dimeric probe was synthesized via a click reaction and labeled with ^99m^Tc to provide a radiotracer (^99m^Tc-HYNIC-D_1_-FA_2_). It was investigated *in vitro* and *in vivo*, including biodistribution, receptor binding in tumor cell culture and imaging in tumor bearing mice with micro-SPECT/CT to verify its tumor targeting ability.

## 2. Results

### 2.1. Chemistry and Radiolabeling

**Scheme 1:**
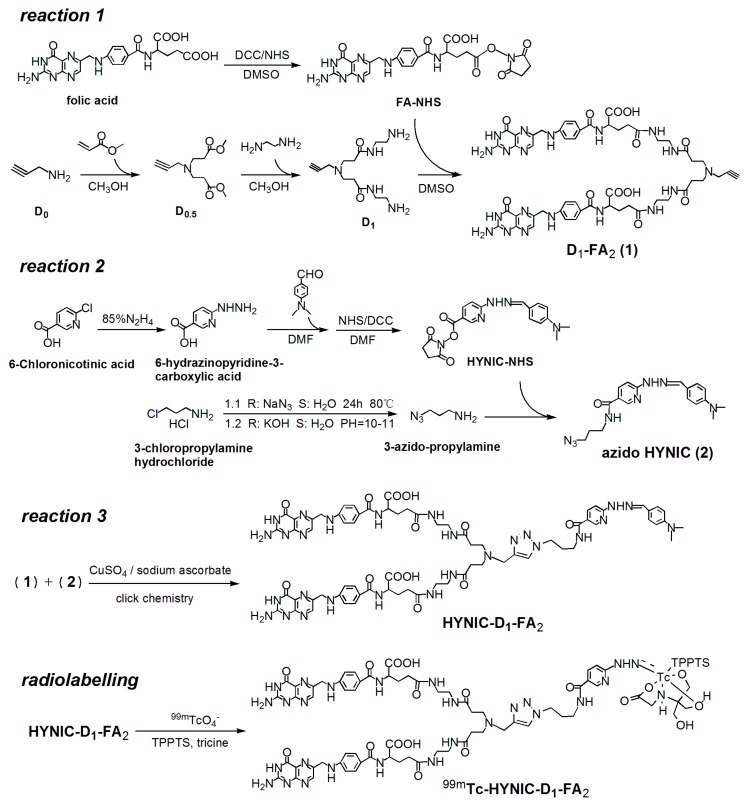
Reaction sequence for the preparation of HYNIC-D_1_-FA_2_ and radiolabeling procedure of ^99m^Tc-HYNIC-D_1_-FA_2_. In our study, the novel folate derivative is composed of four structural components: targeting moiety FA, PAMAM (D_1_), 1,2,3-triazole linkage, and bifunctional chelator.

The general strategy of the synthesis is shown in Scheme 1. FA-NHS and azido HYNIC were synthesized as previously described [11,16]. In order to form folate dimers, a small dendrimer-based platform with unique branching structure (D_1_) was used to attach two targeting moieties (resulting in D_1_-FA_2_) in a well-defined manner. Transitional product D_0.5_ was synthesized from the starting material propynylamine (D_0_) using the same method as the Michael addition of primary amines with methyl acrylate. D_1_ was synthesized from D_0.5_ using the same method used for amidation of methyl ester groups with a large molar excess of ethylenediamine [17,18,19]. The molecular parameters and ^1^H-NMR spectra of PAMAM dendrons are shown in Figure S1. Azide-alkyne click chemistry was employed to conjugate azido HYNIC and D_1_-FA_2_ together to provide HYNIC-D_1_-FA_2_ via an efficient transformation reaction. The corresponding FT-IR spectrum (Figure S2) showed that the characteristic peaks around 2100 cm^−1^ disappeared, indicating the successful click reaction of the alkyne and azide groups in the formation of HYNIC-D_1_-FA_2_. In addition, the UV spectra of folic acid and HYNIC-D_1_-FA_2_ are shown in Figure S3. The characteristic absorption peaks 280 and 363 nm suggested the successful grafting of folic acid in the HYNIC-D_1_-FA_2_, according to its folic acid structure. The presence of HYNIC and folic acid in the HYNIC-D_1_-FA_2_ molecule was confirmed by NMR (Figure S4).

HYNIC is an efficient BFC (bifunctional chelator) for labeling of ^99m^Tc using tricine and TPPTS as coligands [20,21]. High radiochemical yield (>90%) was obtained by using a radiolabeling method. After labeling, a sample of the resulting solution was analyzed and then purified by radio-HPLC. As shown in Figure S5A, the retention time of ^99m^Tc-HYNIC-D_1_-FA_2_ was 14.33 min and the radiochemical purity (RCP) of the purified radiotracer was 98%. The final product was detected by UV and the specific activity was calculated as 2.5 MBq/nmol. The octanol-water partition coefficient (log P) of ^99m^Tc-HYNIC-D_1_-FA_2_ is slightly better than the value of HYNIC-T-FA (shown in Table 1).

### 2.2. In Vitro Experiments

**Figure 1:**
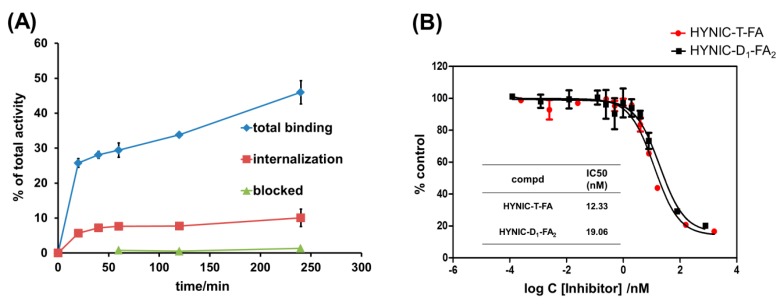
(**A**) The cell binding properties of ^99m^Tc-HYNIC-D_1_-FA_2_ in KB cells (cell total binding, internalization and blocked by excess folic acid, *p* < 0.05). The cell binding was expressed as percentage of total added radioactivity; (**B**) *In vitro* inhibition of ^125^I-tyr-FA binding to FR on KB cells by HYNIC-D_1_-FA_2_.

Time-dependent cell binding and internalization experiments were performed using FR overexpressed KB cells. As shown in Figure 1A, the radiofolates associated with the FRs on KB cells are rapidly accumulated (approaching 50% at 4 h) and approximately 10% of the radiolabeled folate-dimer was internalized. After blocking with excess FA, the cell uptake was reduced drastically. The results of this study confirmed the high specificity of the folate-dimer for the FRs in vitro. The binding curves are shown in Figure 1B and the IC_50_ values of HYNIC-D_1_-FA_2_ and HYNIC-T-FA were calculated as 19.06 nM and 12.33 nM by inhibition experiments using ^125^I-tyr-FA (see Figure S5B).

### 2.3. Biodistribution Study

In order to evaluate the distribution of the radiotracer *in vivo*, biodistribution studies in normal mice (see Table S1) and KB tumor-bearing mice were performed (see Table 2). The animal model was set up in accordance with the previous method. All animal studies were carried out in compliance with the national laws related to the conduct of animal experimentation. ^99m^Tc-HYNIC-D_1_-FA_2_ uptake in the tumor reached a high accumulation (10.16% ± 1.16%ID/g) at 2 h p.i.. Significant uptake intensity was also found in the kidney, and reached a remarkable value at 4 h p.i. (56.69% ± 3.12%ID/g). The uptakes of ^99m^Tc-HYNIC-D_1_-FA_2_ in the other organs, such as heart, liver, stomach, lung and intestine, were kept at a low level. In the blockage study, injection with excess FA leads to an obvious decreased uptake of radiofolate for the FR overexpressed tumor and kidney (2.26% ± 0.19%ID/g and 10.46% ± 0.04%ID/g at 2 h p.i., respectively). PMX was used to increase the tumor-to-kidney ratio of radiofolates based on the literatures [22,23]. Administration of PMX 1 h before the injection of ^99m^Tc-HYNIC-D_1_-FA_2_ significantly reduced the kidney accumulation, whereas the tumor uptake was not changed much.

### 2.4. Static Imaging Study

**Figure 2:**
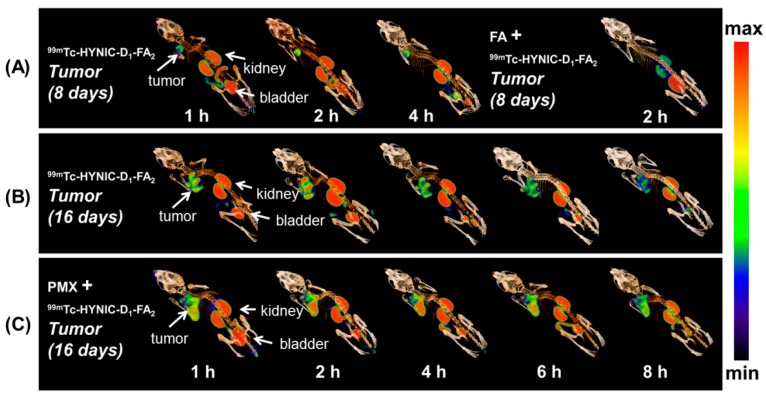
SPECT/CT imaging of xenografted KB-tumor in athymic nude mice. (**A**) 8-days tumor-bearing mice was received ^99m^Tc-HYNIC-D_1_-FA_2_. Blocking study was performed by giving 100 µg FA at 10 min prior to the injection of ^99m^Tc-HYNIC-D_1_-FA_2_; (**B**) 16-days tumor-bearing mice was received ^99m^Tc-HYNIC-D_1_-FA_2_; (**C**) 16-days tumor-bearing mice was received 400 µg PMX at 1 h prior to the injection of ^99m^Tc-HYNIC-D_1_-FA_2_.

The *in vivo* binding specificity of ^99m^Tc-HYNIC-D_1_-FA_2_ to FR was also demonstrated by SPECT imaging in KB tumor-bearing mice. Two tumor stages (8-days and 16-days after tumor cell inoculation) were imaged by using ^99m^Tc-HYNIC-D_1_-FA_2_. The images of 8-days tumors are shown in Figure 2A. The probe was effective against early-stage cancer even at a small size, which increases the chance of early diagnosis and treatment of tumors. By pre-administration of excess free FA (100 µg FA was injected at 10 min prior to the radiotracer) via a lateral tail vein, the uptake in FR overexpressed kidney and tumor were significantly reduced. For the 16-days tumor, the KB tumor and kidney were clearly visualized at all time points (Figure 2B), this result was consistent with the biodistribution data. A reduced kidney uptake and increased tumor-to-kidney ratio were obtained as expected by pre-administration of PMX (Figure 2C).

**Figure 3:**
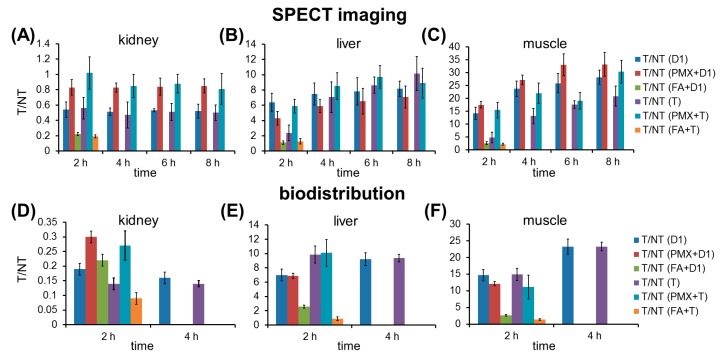
T/NT ratios of ^99m^Tc-HYNIC-D_1_-FA_2_ and ^99m^Tc-HYNIC-T-FA in SPECT imaging (**A**–**C**) and biodistribution studies (**D**–**F**). Uptakes of organs were calculated by drawing ROIs on the SPECT/CT images. D1 = ^99m^Tc-HYNIC-D_1_-FA_2_; T = ^99m^Tc-HYNIC-T-FA. PMX block study: PMX (400 µg) 1 h prior to ^99m^Tc-HYNIC-D_1_-FA_2_. FA block study: Folic acid (100 µg) 10 min prior to ^99m^Tc-HYNIC-D_1_-FA_2_.

T/NT ratios of ^99m^Tc-HYNIC-D_1_-FA_2_ and previously reported ^99m^Tc-HYNIC-T-FA [11] are compared in Figure 3. In SPECT imaging, ^99m^Tc-HYNIC-D_1_-FA_2_ has much higher tumor-to-muscle ratio than that of ^99m^Tc-HYNIC-T-FA, while their tumor-to-kidney ratios were almost same. In the biodistribution results, dimeric ^99m^Tc-HYNIC-D_1_-FA_2_ did not show increased T/NT ratios when compared with monomeric ^99m^Tc-HYNIC-T-FA. Both of tumor-to-kidney ratios of these two radiofolates could be obviously increased by pre-administration of PMX.

### 2.5. Dynamic Imaging Study

**Figure 4:**
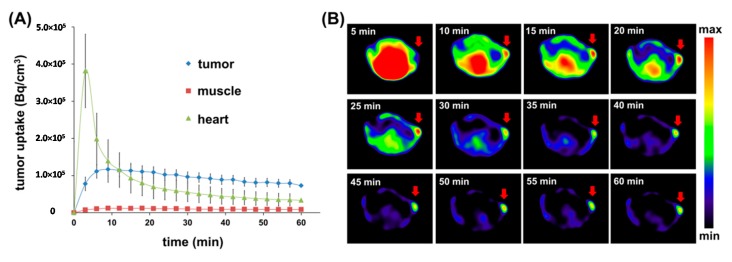
(**A**) Time-activity curves (TACs) of ^99m^Tc-HYNIC-D_1_-FA_2_ in KB tumor-bearing mice derived from the 60 min dynamic SPECT/CT scan; (**B**) Transversal tumor SPECT images at different time points of image acquisition.

For semiquantitative dynamic SPECT imaging, as shown in Figure 4A, from early time points (15 min p.i.), a relatively high accumulation of radioactivity was observed in the tumor, and significant retention was also indicated over the time of investigation with low muscle uptake. Fast clearance from the heart (blood) was observed during the earlier 15 min p.i. and then the uptake lower than that of tumor afterward. Figure 4B shows SPECT images of corresponding transversal sections of KB tumor at different detection times. The nonspecific retentions of radioactivity in tumor surrounding tissues were decreased obviously over time, leading to a clear outline of tumor (marked with an arrow).

## 3. Discussion

With the development of molecular imaging, diagnosis as well as therapeutic methods for diseases are becoming more and more exact, credible and rapid. Nuclear medicine plays a crucial role in the development of molecular imaging and precision medicine, depending on different types of molecular probes and innovative techniques.

SPECT offers unique capabilities of easier preparation and much lower costs. An efficient ternary ligand system (HYNIC, tricine and TPPTS) has been used in ^99m^Tc-labeling of biomolecules. In this study, a lyophilized kit formulation of HYNIC-D_1_-FA_2_ has been developed to simplify the radiolabeling and purification procedure. In this situation, a one-pot synthesis of ^99m^Tc-HYNIC-D_1_-FA_2_ has significant advantages over the corresponding ^18^F-labeled FA derivatives and should be easily acceptable for clinical application. In recent years, various folate radiotracers have been reported increasingly [11,21,24,25,26]. Compared with other reported monomeric probes (as shown in Table 3), ^99m^Tc-HYNIC-D_1_-FA_2_ performs much better in tumor uptake value and T/NT ratio. All of these comprehensive advantages lead to its potential as folate radiotracer candidate for tumor detection.

Many radiolabeled multimeric cyclic RGD peptides have been used in α_v_β_3_-positive tumor imaging by SPECT and PET. Construction of multimers has proved to be an effective approach to improve the tumor uptake. Although some previous studies have shown that radiolabeled FR-targeting PAMAM dendrimers can be used in live animal micro-SPECT imaging studies, there are so many primary amine groups on the branched surface that are difficult to control the number of BFCs and targeting precursors conjugated to the PAMAM dendrimer. Besides uncertain molecular structure, folate-PAMAM dendrimers with large molecular weight are associated with cytotoxicity and excessive liver retention, which limit their further applications *in vitro* and *in vivo* [27,28].

Different from the multimeric concept based on PAMAM dendrimers, here we use a small alkyne-functionalized molecule with two branches to build a small system to link two identical targeting elements. Based on this design, we believe that if the distance of the FA in HYNIC-D_1_-FA_2_ is suitable, the two FAs entities will bind to FRs on the cell surface. In this case, weak ligand-receptor interactions would be enhanced. If simultaneous FRs binding is difficult, the FA concentration is still “locally enriched” in the vicinity of neighboring FR sites once the first FA element is bound, which is similar to the concept of multimeric cyclic RGD. In our study, the results showed that this approach can improve the radiotracer’s FR-targeting capability and minimize its accumulation in normal organs. Both *in vitro* experiments and SPECT studies showed that the radiolabeled HYNIC-D_1_-FA_2_ had excellent tumor uptake and better ratios of tumor-to-muscle and tumor-to-liver than that of monomeric FR-targeting probe due to its enhanced uptake in tumor but decreased uptake in muscle and liver.

In this study, as shown in Figure 3, the SPECT imaging and biodistribution results of ^99m^Tc-HYNIC-D_1_-FA_2_ were compared to previously published data of ^99m^Tc-HYNIC-T-FA using KB-tumor bearing mice [11]. In imaging study, ^99m^Tc-HYNIC-D_1_-FA_2_ had a tumor-to-kidney ratio which is equivalent to ^99m^Tc-HYNIC-T-FA. Application of PMX effectively raise the tumor-to-kidney and tumor-to-muscle ratios for both dimer and monomer at all observation time points. In blocked study, tumor-to-muscle ratio was reduced to 2.59 after injection of free FA, supporting FR-specific binding of ^99m^Tc-HYNIC-D_1_-FA_2_. From the perspective of tumor-to-muscle ratio, ^99m^Tc-HYNIC-D_1_-FA_2_ obtained a higher score and showed better tumor image contrast. This advantage was reflected in biodistribution study. While, it's important to note that in order to ensure minimal time used for imaging, SPECT/CT studies required higher amounts of radio folate (as well as folate mass) than biodistribution. Consequently, we could present only a qualitative comparison of the data from the biodistribution and SPECT/CT studies [29,30].

## 4. Materials and Methods

### 4.1. Reagents and Materials

All chemicals obtained commercially were used without further purification. *N*-Tris-(hydroxymethyl)-methylglycine (tricine), trisodium triphenylphosphine-3,3,3′′-trisulfonate (TPPTS), *N*-hydroxylsuccinimide (NHS) and dicyclohexylcarbodiimide (DCC) and other reagents were purchased from J & K Chemical Ltd. (Beijing, China). The eluent Na^99m^TcO_4_ were obtained from Zhongshan Hospital Affiliated of Xiamen University. The radioactivity was counted with γ-counter (WIZARD 2480, Perkin-Elmer, Waltham, MA, USA) and CRC-25R Dose Calibrators (CAPIN-TEC Inc., Ramsey, NJ, USA). SPECT imaging studies were performed using a nanoScan-SPECT/CT scanner (Mediso, Budapest, Hungary). The PET/CT study was performed using Siemens Inveon device (Siemens Corp., Berlin, Germany). ^1^H-NMR spectra were measured on a Bruker (400 MHz) spectrometer (Bruker, Karlsruhe, Germany). FT-IR spectroscopy (AVATAR 360, Nicolet Company, Madison, WI, USA) was used to analyze reaction progress. The UV spectroscopy was performed using a microplate spectrophotometer (Multiskan GO, Thermo Fisher, Waltham, MA, USA). Mass spectra (MS) were recorded using a Bruker Apex IV FTM instrument. Kit formulations were lyophilized using a lyophilizer (Freezone Triad 2.5L, LABCONCO, Kansas, MO, USA). Dionex Ulti-Mate 3000 HPLC (Thermo Scientific, Waltham, MA, USA) with flow-counter radioactivity detector (BioScan, Poway, CA, USA) was used to test the radiochemical purity. The mobile phase is presented below: A: 90% NH_4_HCO_3_ (0.05 mol/L)/10% CH_3_OH, B: 100% CH_3_OH; 0–10 min, B: 5%–50%; 10–20 min, B: 50%–50%; 20–30 min, B: 50%–5%; flow rate: 1 mL/min.

### 4.2. Synthetic Route to HYNIC-D_1_-FA_2_

#### 4.2.1. Reaction 1

FA-NHS and propargyl-PAMAM dendron D_1_ were successfully synthesized and the corresponding D_1_-FA_2_ was prepared by amidation.

##### Preparation of FA-NHS

Folic acid (3 g, 6.8 mmol), NHS (940 mg, 8.2 mmol) and DCC (1.68 g, 8.2 mmol) were dissolved in DMSO (50 mL) under vigorous stirring and stirred at room temperature for 24 h in the dark. The byproduct was removed by filtration to give a reddish-brown filtrate containing FA-NHS.

##### Preparation of Propargyl-PAMAM Dendrons (D_1_)

Propargyl-PAMAM dendrons with primary amine terminal groups (D_1_) were synthesized according to the procedure similar to that reported by Lee *et al*. [18]. A solution of propargylamine (indicated as D_0_, 5 g, 90.7 mmol) in methanol (30 mL) was added dropwise over 30 min to a cooled (ice-water bath) and stirred solution of methyl acrylate (78 g, 907.0 mmol) in methanol (80 mL). The resulting solution was stirred for 1 h at 0 °C and then reacted at room temperature under a nitrogen atmosphere for a further 48 h. The reaction solvent was removed under vacuum to give the dendron D_0.5_ as yellow oil. ^1^H-NMR (CDCl_3_): δ3.67 (s, 6H), 3.42 (s, 2H), 2.85–2.81 (t, 4H), 2.48–2.44 (t, 4H), 2.26–2.25 (d, 1H). ESI-MS: *m*/*z* calcd for C_11_H_17_NO_4_: 227.3, found 228.3 [M + H]^+^. Next a solution of D_0.5_ (5 g, 21.9 mmol) in methanol (30 mL) was added dropwise to a cooled (ice-water bath) and stirred solution of 1,2-diaminoethane (53 g, 876 mmol) in methanol (80 mL) over 30 min. The resulting solution was stirred for 1 h at 0 °C and then reacted at room temperature under a nitrogen atmosphere for a further 72 h. The reaction solution was removed under reduced pressure using a rotary evaporator, to give the dendron D_1_ as a yellow oil with two primary amine terminal groups. ^1^H-NMR (D_2_O): δ3.35 (s, 2H), 3.18–3.15 (t, 4H), 2.80–2.76 (t, 4H), 2.65–2.64 (t, 4H), 2.62 (s, 1H), 2.39–2.35 (t, 4H). ESI-MS: *m*/*z* calcd for C_3_H_9_N_4_: 283.6, found 284.9 [M + H]^+^.

##### Preparation of D_1_-FA_2_

D_1_ (0.85 g, 3 mmol) and DIPEA (1.1 g, 8.4 mmol) were added dropwise to the filtrate containing FA-NHS. The mixture was stirred for 2 days in the dark at room temperature to ensure sufficient reaction of FA-NHS and D_1_. In order to remove unreacted raw materials, the rough product was dissolved in DMSO and dialyzed (cellulose membrane with 500 MWCO) against DMSO for 3 days. Then the solution was dropped into ether. The precipitate was collected by filtration, and then dried under vacuum to afford the crude product.

#### 4.2.2. Reaction 2

Azido HYNIC were synthesized according to a procedure similar to that reported in our previous article [11].

##### Preparation of 3-azidopropyl-1-amine

To a solution of 3-chloropropylamine hydrochloride (5.0 g, 38.5 mmol) in water (15 mL) was slowly added a solution of NaN_3_ (7.5 g, 115.3 mmol) in water (30 mL). The resulting solution was heated at 80 °C for 18 h. After cooling to room temperature, about 1/2 to 2/3 of the water was removed under vacuum. The remaining residue was diluted with ether (50 mL). This biphasic mixture was cooled in an ice bath for 5 min and KOH (2.0 g) was added. The phases were separated and the aqueous phase was extracted with diethyl ether (2 x 50 mL). The organic layers were combined, dried with Na_2_SO_4_, and concentrated to give the 3-azidopropylamine as light yellow oil (2.9 g, 29.0 mmol) in 75% yield. ^1^H-NMR (CDCl_3_): δ3.39–3.36 (t, 2H), 2.82–2.79 (t, 2H), 1.77–1.70 (m, 2H), 1.45 (bs, 2H). ESI-MS: *m*/*z* calcd for C_3_H_9_N_4_: 100.1, found 101.1 [M + H]^+^.

##### Preparation of azido HYNIC

6-Chloronicotinic acid (5 g; 31.7 mmol) was added to 80% hydrazine hydrate (22 mL; 584.6 mmol) and the resulting solution was heated at 100 °C for 6 h. Then the reaction mixture was concentrated to dryness to afford a white solid. The solid was dissolved in water (50 mL). The pH was adjusted to 5.5 by addition of concentrated hydrochloric acid, and a yellow precipitate was formed. The precipitate was collected by filtration, washed with 95% ethanol and ether and dried under high vacuum to afford 3.6 g of 6-hydrazinopyridine-3-carboxylic acid in a yield of 75%.

6-Hydrazinopyridine-3-carboxylic acid (3 g, 19.6 mmol) was dissolved in DMF (50 mL) and 4-dimethylaminobenzaldehyde (3.2 g, 21.9 mmol) was then added. The reaction mixture was allowed to react at room temperature. After being stirred for 8 h, the resulting solution was mixed with NHS (2.3 g, 20 mmol) and DCC (8.5 g, 41 mmol) and stirring was continued for 24 h. The reaction mixture was filtered to give a reddish-brown filtrate containing 6-BOC-HYNIC-NHS.

3-Azidopropyl-1-amine (2.9 g, 29.0 mmol) and DIPEA (2.53 g, 19.6 mmol) were added dropwise to the filtrate containing 6-BOC-HYNIC-NHS. The mixture was stirred for 2 days at room temperature, and then concentrated to dryness to give a yellow residue. The residue was dissolved in ethyl acetate (50 mL). The desired product was separated by filtration and dried under vacuum to give 4.2 g of a yellow solid (yield 71%). The azido HYNIC was used for the next reaction without further purification. ^1^H-NMR (DMSO-*d*_6_): δ8.59 (s, 1H), 8.32 (s, 1H), 8.05–8.04 (d, 1H), 7.95 (s, 1H), 7.49 (d, 2H), 7.15–7.14 (d, 1H), 6.75 (d, 2H), 5.57–5.56 (d, 2H), 3.42–3.39 (q, 2H), 2.95 (s, 6H), 1.77–1.73 (m, 2H), 1.51–1.48 (t, 2H). ESI-MS: *m*/*z* calcd for C_18_H_22_N_8_O: 366.5, found 367.5 [M + H]^+^.

#### 4.2.3. Reaction 3

HYNIC-D_1_-FA_2_ was prepared by a transformation which has been denoted as a “click reaction”.

##### Preparation of HYNIC-D_1_-FA_2_

D_1_-FA_2_ (2.26 g, 2 mmol) and azido HYNIC (734 mg, 2 mmol) were dissolved in *t*-BuOH/H_2_O (1:1, 30 mL). The mixture was stirred under a nitrogen atmosphere. Then CuSO_4_·5H_2_O (50 mg, 0.2 mmol) and sodium ascorbate (80 mg, 0.4 mmol) were added. The reaction flask was sealed and the reactions were carried out at 70 °C for 1 h in the dark. NaOH (1 mol/L) was added dropwise until the solutions were clear, followed by cooling the mixture to room temperature, and filtration. The product was precipitated by adjusting the pH to 3 with HCl (1 mol/L). The resulting suspension was centrifuged and the pale yellow supernatant decanted. The solid was dried under vacuum to give the HYNIC-D_1_-FA_2_ as a brown powder. In order to remove unreacted HYNIC and folate moieties, the crude product were further purified by HPLC. ESI-MS: *m*/*z* calcd for C_18_H_22_N_8_O: 1495.7, found 1496.7 [M + H]^+^.

### 4.3. Radiolabeling

The labeling method was as follows: to a 10 mL vial was added tricine solution (0.5 mL, 80 mg/mL in saline), HYNIC-D_1_-FA_2_ solution (100 μL, 1 mg/mL in PBS, pH 7.4), TPPTS (0.2 mL, 5 mg/mL in saline), SnCl_2_ solution (20 μL, 2 mg/mL) in 0.1 N HCl and about 1 mL of ^99m^TcO_4_^−^ (370 MBq) in saline. The vial was heated at 100 °C for 30 min in a heating module. After cooling to room temperature, a sample of the resulting solution was purified and analyzed by Sep-Pak C18 cartridge and radio-HPLC. In further experiments, a kit formulation was developed for preparation of ^99m^Tc-HYNIC-D_1_-FA_2_ using this ternary ligand system.

### 4.4. Octanol/Water Partition Coefficient

To determine the hydrophilicity of ^99m^Tc-HYNIC-D_1_-FA_2_, the partition coefficient (expressed as log P) was measured following the method: 100 μL radiotracer solution was diluted with 2.9 mL PBS (0.05 mol/L, pH = 7.4) and 3 mL 1-octanol. After shaking for 3 min, the mixture was centrifuged at 6000 rpm for 5 min. The counts of 100 μL organic layer and 100 μL inorganic layer were determined by a gamma counter, respectively. The following equation was used to calculate log P: P = (activity in octanol phase—background activity)/(activity in aqueous phase—background activity). All the experiments were performed with triplicate samples and reported as mean ± SD.

### 4.5. In Vitro Experiments

The cell culture process has been described in a previous article [11]. For cell uptake study, 0.3 μCi ^99m^Tc-HYNIC-D_1_-FA_2_ in 100 μL folate-free RPMI medium was added to each well and incubated at 37 °C. After incubation, the medium was removed and the cells were gently washed three times with cold PBS (pH 7.4, including 0.2% bovine serum albumin) to determine total radiofolate uptake. Cellular internalization of the ^99m^Tc-HYNIC-D_1_-FA_2_ was assessed by additionally washing with 500 μL stripping buffer (pH 4.0, including 0.2% bull serum albumin) two times. The stripping buffer was then transferred to tubes. Finally, the cells were lysed by treatment with 1 M NaOH for 5–10 min and transferred to tubes. The blocking studies were performed by addition of free folic acid solution (10 μL, 1 mg/mL). Then, 100 μL radiotracer was added, and the well plates were incubated at 37 °C for 1 h, 2 h and 4 h, respectively. After incubation, the medium were removed, and the cells were rinsed with cold PBS. Finally, the cells were lysed by treatment with 1 M NaOH for 5–10 min and transferred to tubes.

### 4.6. Biodistribution Study

The biodistribution of ^99m^Tc-HYNIC-D_1_-FA_2_ was evaluated in normal BALB/c mice and KB tumor-bearing mice (18–20 g, female), and the mice were maintained on a folate-deficient diet for 5 days before the study. 25 BALB/c mice were randomly divided into five groups and 20 KB tumor-bearing mice were randomly divided into four groups, each of which had five animals. Approximately 37 kBq of the purified radiotracer was administered via a lateral tail vein. Then the mice (*n* = 5) were sacrificed at different time points. The interested tissues and organs were excised, weighed and counted in a gamma counter. The results were calculated as a percentage of the injected dose per gram of tissue (% ID/g). In order to confirm that the ^99m^Tc-HYNIC-D_1_-FA_2_ had specific receptor binding, mice were performed by injection with free folic acid (100 μg/mouse) as blocking agent 10 min prior to the radiotracer injection. In order to reduce undesired accumulation in the kidneys, antifolate PMX was used to reduce accumulation in the kidneys. The PMX was injected via a lateral tail vein (400 μg/100 μL) 1 hour prior to the radiotracer injection. Animals were sacrificed by decapitation for biodistribution using the same procedure above 2 h after radiotracer injection.

### 4.7. Static SPECT Imaging Study

The imaging study was performed using a nanoscan SPECT/CT preclinical imager (Mediso). For static SPECT imaging, each mouse was injected with 18.5 MBq/200 μL ^99m^Tc-HYNIC-D_1_-FA_2_ via a lateral tail vein. Anesthesia was induced with isoflurane and spontaneous breathing was maintained during the scan procedure. CT data were acquired using an X-ray voltage biased to 50 kVp with a 670 μA anode current, and the projections were 720°. SPECT acquiring parameters were as follows: 140 keV energy peak for ^99m^Tc, window width of 20%, matrix of 256 × 256, medium zoom, and frame: 30 s. The static pinhole SPECT imaging was performed at different time point to investigate the FR binding properties of ^99m^Tc-HYNIC-D_1_-FA_2_ *in vivo*. As for the blocking study, 100 μg of excess folic acid (1 mg/mL folic acid in PBS) was administered via intravenous injection 10 min prior to the radiotracer injection. The imaging scans of the blocking study were performed at 2 h p.i. In order to measure the tumor-to-kidney ratio, the antifolate drug pemetrexed (PMX, 400 μg in 100 μL PBS) was injected via intravenous injection, 1 h before the radiotracer.

### 4.8. Dynamic SPECT Imaging Study

For dynamic SPECT imaging, the indwelling needle was prepared before injecting the mouse with 18.5 MBq/200 μL ^99m^Tc-HYNIC-D_1_-FA_2_ via a lateral tail vein. Dynamic semiquantitative SPECT/CT imaging was performed for 60 min (12 × 300 s) after injection, and anesthesia was induced with isoflurane during the scan procedure. TACs were derived by drawing ROIs on the SPECT/CT images.

## 5. Conclusions

In this study, we report a novel FR-targeting dimeric FA conjugate labeled with ^99m^Tc as a potential SPECT radiopharmaceutical for clinical applications. The combination reaction of the labeling entity and the targeting precursor was carried out via a click chemistry approach. The new dimeric probe can be produced by a reliable radiosynthesis using tricine and TPPTS as coligands. In vitro, *ex vivo* and *in vivo* studies shows that ^99m^Tc-HYNIC-D_1_-FA_2_ has high affinity and specificity to the FR, and might be a promising candidate for further clinical translational study in FR-positive cancer and imaging inflammatory diseases with SPECT.

## Figures and Tables

**Table 1:** Molecular parameters of HYNIC-T-FA and HYNIC-D_1_-FA_2_.

Derivative	FA/HYNIC	Molecular Formula	Mw	Log P
HYNIC-T-FA	1/1	C_40_H_44_N_16_O_6_	844.4	−2.40 ± 0.17
HYNIC-D_1_-FA_2_	2/1	C_69_H_81_N_27_O_13_	1495.7	−2.52 ± 0.13

**Table 2:** The biodistribution results of ^99m^Tc-HYNIC-D_1_-FA_2_ in KB tumor-bearing mice (%ID/g, mean ± SD, *n* = 5).

Tissues	Post-Injection Time			
	2 h	4 h	2 h w/ PMX *	2 h w/ FA **
Heart	1.11 ± 0.18	0.90 ± 0.08	0.75 ± 0.10	0.82 ± 0.12
Liver	1.45 ± 0.56	1.01 ± 0.11	1.23 ± 0.14	0.88 ± 0.10
Lung	1.59 ± 0.62	0.97 ± 0.11	1.05 ± 0.31	1.27 ± 0.18
Kidney	54.09 ± 2.21	56.69 ± 3.12	28.07 ± 2.42	10.46 ± 0.04
Spleen	0.95 ± 0.05	0.78 ± 0.31	0.85 ± 0.18	0.99 ± 0.14
Stomach	1.03 ± 0.11	0.65 ± 0.09	0.73 ± 0.03	0.46 ± 0.03
Bone	1.11 ± 0.07	0.98 ± 0.07	0.76 ± 0.16	0.91 ± 0.21
Muscle	0.69 ± 0.04	0.40 ± 0.07	0.70 ± 0.10	0.86 ± 0.12
Intestines	0.80 ± 0.12	0.63 ± 0.07	0.63 ± 0.11	0.76 ± 0.16
Blood	0.66 ± 0.19	0.58 ± 0.04	0.66 ± 0.16	0.96 ± 0.20
Tumor	10.16 ± 1.16	9.30 ± 0.91	8.47 ± 0.45	2.26 ± 0.19
Tumor/Muscle	14.72	23.25	12.10	2.63
Tumor/Kidney	0.19	0.16	0.30	0.22

* 400 µg PMX was injected at 1 h prior to ^99m^Tc-HYNIC-D_1_-FA_2_; ** 100 µg FA was injected at 10 min prior to ^99m^Tc-HYNIC-D_1_-FA_2_.

**Table 3:** Biodistribution data (4 h p.i.) of radiofolates in athymic nude mice bearing KB tumor xenografts (%ID/g, mean ± SD).

	Blood	Muscle	Tumor	Kidneys	Liver
^111^In-DTPA-folate [24]	0.03 ± 0.02	0.71 ± 0.26	2.9 ± 0.9	25.0 ± 6.0	0.64 ± 0.23
^99m^Tc-DTPA-folate [24]	0.19 ± 0.05	0.70 ± 0.14	2.9 ± 0.8	21.0 ± 3.0	1.05 ± 0.30
^99m^Tc(CO)_3_-DTPA-folate [25]	0.37 ± 0.02	2.40 ± 0.10	3.3 ± 0.2	47.0 ± 5.0	7.60 ± 0.50
^99m^Tc(HYNIC-lys-pteroyl)(tricine/TPPTS) [21]	0.31 ± 0.07	0.98 ± 0.17	7.9 ± 1.4	88.6 ± 9.2	2.08 ± 0.18
^99m^Tc(HYNIC-NHHN-FA)(tricine/TPPMS) [26]	0.06 ± 0.00	0.17 ± 0.07	0.19 ± 0.05	3.6 ± 1.4	0.14 ± 0.07
^99m^Tc(HYNIC-NHHN-FA)(tricine/TPPTS) [26]	0.30 ± 0.07	0.81 ± 0.23	9.8 ± 1.7	114.9 ± 8.1	0.60 ± 0.13
^99m^Tc-HYNIC-T-FA [11]	0.37 ± 0.05	0.35 ± 0.11	8.14 ± 0.5	57.72 ± 4.5	0.85 ± 0.11
^99m^Tc-HYNIC-D_1_-FA_2_	0.58 ± 0.04	0.40 ± 0.07	9.30 ± 0.91	56.69 ± 3.12	1.01 ± 0.11

## Supplementary Materials

Supplementary materials can be accessed at: http://www.mdpi.com/1420-3049/21/6/817/s1.

## Abbreviations

The following abbreviations are used in this manuscript:

FA	folic acid
FR	folate receptor
HYNIC	2-hydrazinonicotinic acid
BFC	bifunctional chelator
DMSO	Dimethyl Sulphoxide
DCC	*N*,*N*′-Dicyclohexylcarbodiimide
NHS	*N*-Hydroxysuccinimide
Tricine	*N*-tris-(hydroxymethyl)-methylglycine
TPPTS	trisodiumtriphenylpho-sphine-3,30,3″-trisulfonate
PEG	poly-(ethylene glycol)
PAMAM	polyamidoamine
PMX	pemetrexed
SPECT	single photon emission computed tomography
RGD	cyclic arginine-glycine-aspartic acid peptides
^99m^Tc	technetium-99m
MRI	magnetic resonance imaging
PB	phosphate buffer
ITLC-SG	Instant thin-layer chromatography silica gel strips
TLC	thin-layer chromatography
RCP	radiochemical purity
SA	specific activity
%ID/g	percentage of injected dose per gram
TACs	time-activity curves
ROIs	regions of interests
T/NT	tumor-to-nontarget tissue
3D	three-dimensional
HPLC	High Performance Liquid Chromatography
CT	computed tomography
^18^F-FDG	^18^F-fluorodeoxyglucose
p.i.	post-injection time
