# Supplementary material for: Synthesis and Evaluation of 99mTc-Labeled Dimeric Folic Acid for FR-Targeting
Source: Molecules. 2016 Jun 22;21(6):817. doi: 10.3390/molecules21060817 (PMC6274367; doi:10.3390/molecules21060817)
Supplement: Supplementary file 1 [file molecules-21-00817-s001.pdf]

Supplementary Materials: Synthesis and Evaluation of ^{99m}Tc-Labeled Dimeric Folic Acid for FR-Targeting

Zhide Guo, Mengna Gao, Manli Song, Changrong Shi, Pu Zhang, Duo Xu, Linyi You, Rongqiang Zhuang, Xinhui Su, Ting Liu, Jin Du and Xianzhong Zhang

(A)

Generation	NH_2 Number	Molecular Formula	Determined Mw	Yield (%)
D_0	1	C_3H_5N	55.3	/
$D_{0.5}$	0	$C_{11}H_{17}NO_4$	227.3	94.2
D_1	2	$C_{13}H_{25}N_5O_2$	283.6	96.5
Azido-propylamine	0	$C_3H_8N_4$	100.1	96.2

(B)

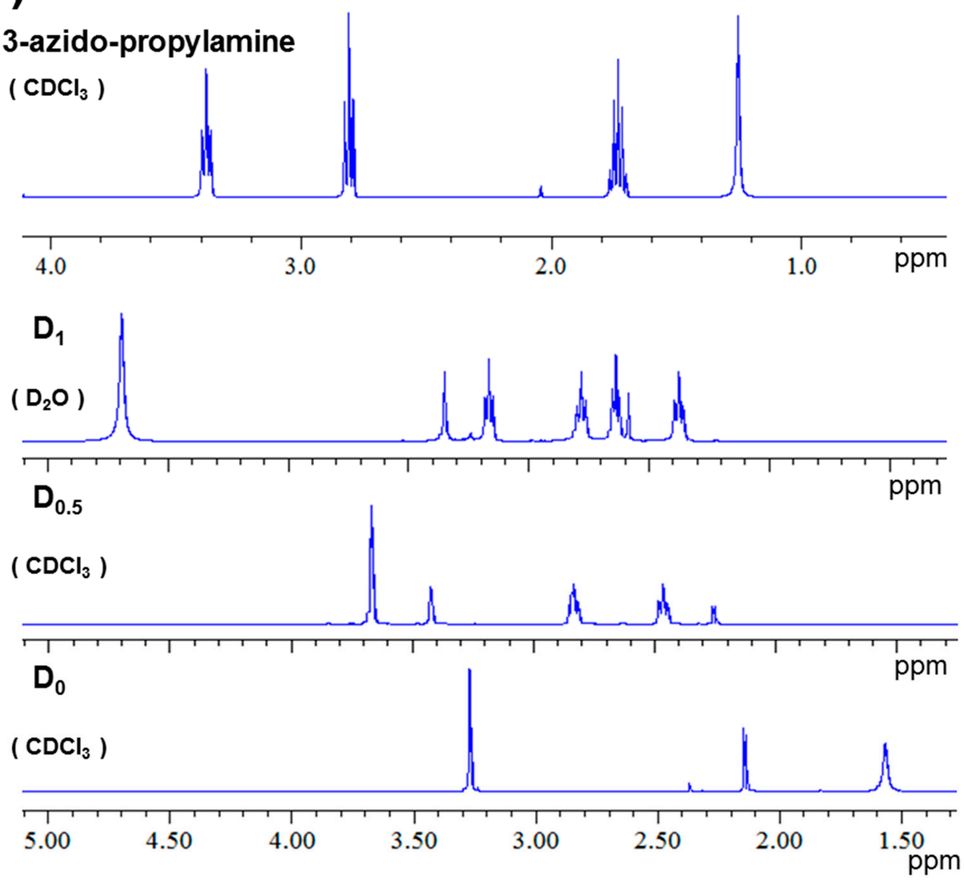

Figure S1. (**A**) Molecular parameters of PAMAM dendrons and propargylamine; (**B**) ^{1}H-NMR spectra of the PAMAM dendrons and propargylamine.

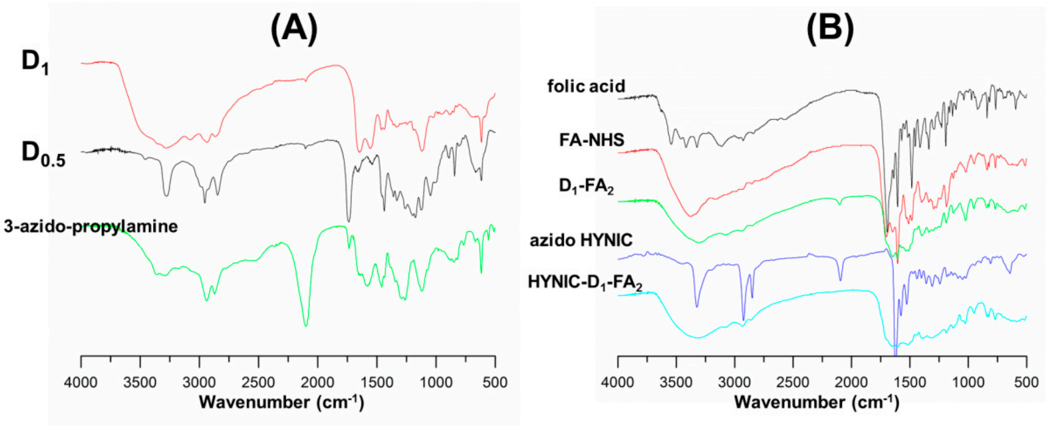

Figure S2. (**A**) FT-IR spectra of D_1, $D_{0.5}$ and 3-azidopropylamine; (**B**) FT-IR spectra of folic acid, FA-NHS, D_1-FA_2, azido HYNIC and HYNIC-D_1-FA_2.

As shown in Figure S2, the absorption recorded at 2100 cm^{-1} revealed the presence of alkyne groups in $D_{0.5}$, D_1 and D_1-FA_2. Similarly, the characteristic absorption of the azide groups in 3-azido-propylamine and azide-functionalized HYNIC was also observed at 2100 cm^{-1}. Moreover, neither the band corresponding to azide nor alkyne was observed in the final product, indicating the successful incorporation of the alkyne and azide groups in the formation of HYNIC-D_1-FA_2.

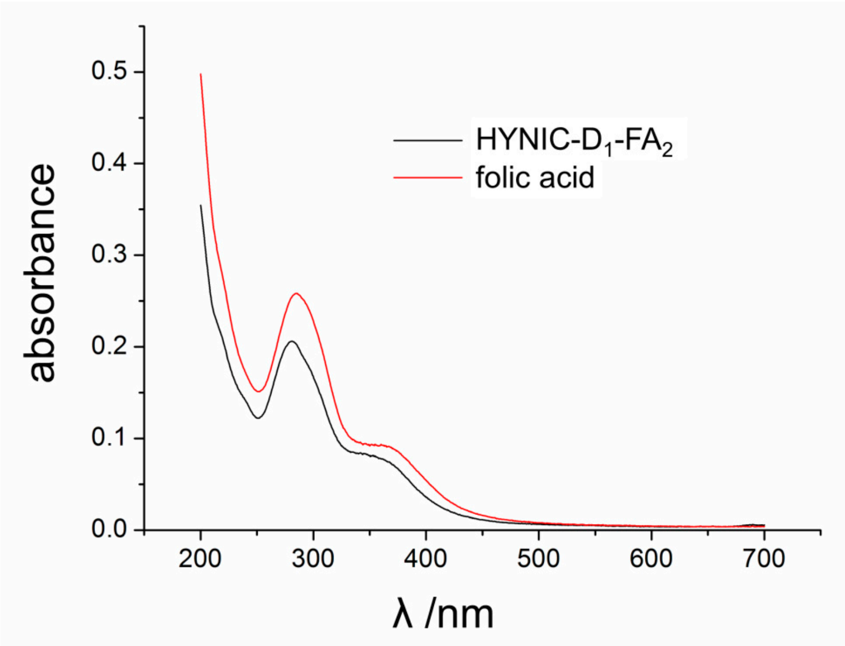

Figure S3. UV analysis of folic acid and HYNIC-D_1-FA_2.

Comparing the UV absorption peaks of folic acid and HYNIC-D_1-FA_2 (288 and 363 nm), the peak positions suggested the successful grafting of folic acid in the HYNIC-D_1-FA_2.

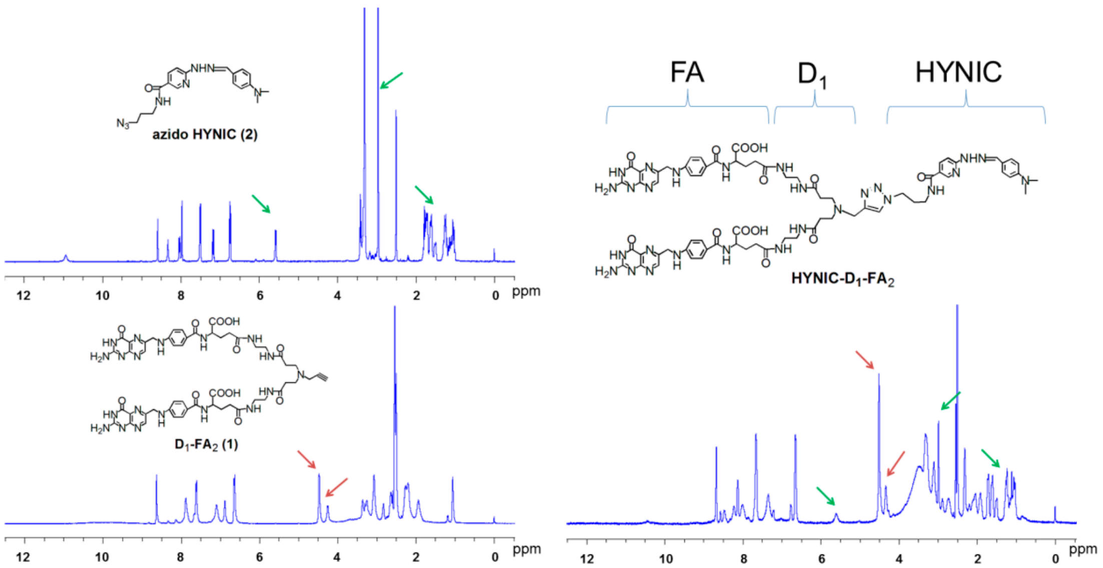

Figure S4. ^{1}H-NMR spectra of the azido HYNIC, D_1-FA_2 and HYNIC-D_1-FA_2.

Collectively, the results from the ^{1}H-NMR spectra for defining molecular structure clearly demonstrated the successful synthesis of the HYNIC-D_1-FA_2 conjugate using our experimental protocol.

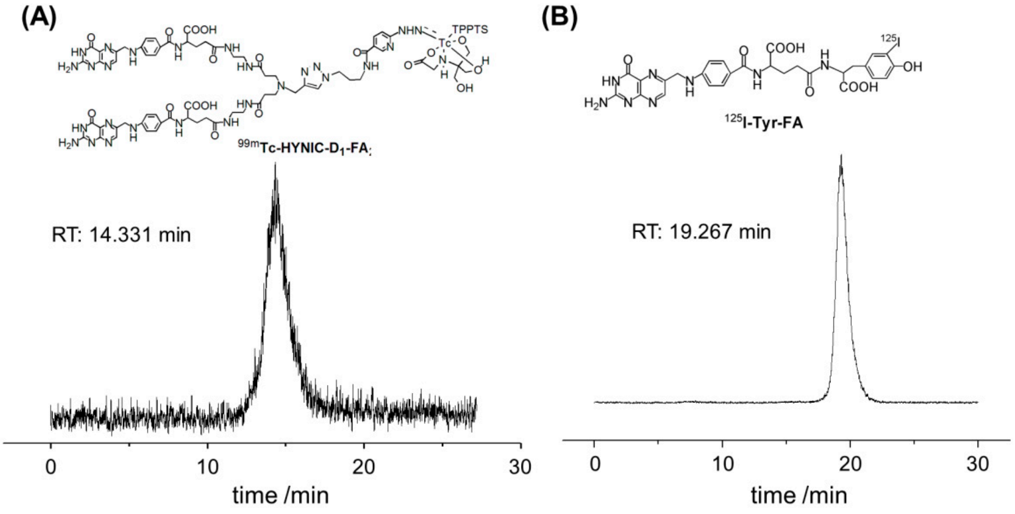

Figure S5. HPLC chromatograms of ^{99m}Tc-HYNIC-D_1-FA_2 and ^{125}I-Tyr-FA.

As shown in Figure S5, the retention times (RT) of ^{99m}Tc-HYNIC-D_1-FA_2 and ^{125}I-Tyr-FA were 14.331 and 19.267 min.

Table S1. The biodistribution result of ^{99m}Tc-HYNIC-D_1-FA_2 in normal mice (%ID/g, mean ± SD, *n* = 5).

**Tissues**	**Post-Injection Time**				
	**5 min**	**1 h**	**2 h**	**4 h**	**1 h-block** [1]
Heart	3.48 ± 0.08	0.90 ± 0.17	0.49 ± 0.06	0.41 ± 0.06	0.41 ± 0.11
Liver	4.13 ± 0.89	1.08 ± 0.18	0.67 ± 0.12	0.55 ± 0.04	0.58 ± 0.12
Lung	6.67 ± 0.56	1.14 ± 0.22	0.67 ± 0.09	0.35 ± 0.04	1.00 ± 0.19
Kidney	56.05 ± 6.92	81.04 ± 4.19	94.78 ± 8.01	111.30 ± 22.25	12.35 ± 2.90
Spleen	1.86 ± 0.27	0.50 ± 0.18	0.22 ± 0.05	0.01 ± 0.002	0.39 ± 0.18
Stomach	1.19 ± 0.11	1.69 ± 0.30	0.89 ± 0.31	0.94 ± 0.13	0.86 ± 0.20
Bone	2.91 ± 0.33	0.80 ± 0.15	0.26 ± 0.09	0.26 ± 0.09	0.77 ± 0.03
Muscle	2.22 ± 0.25	0.48 ± 0.12	0.29 ± 0.04	0.19 ± 0.05	0.34 ± 0.10
Intestines	3.84 ± 0.26	0.65 ± 0.10	0.53 ± 0.07	0.25 ± 0.07	0.78 ± 0.29
Blood	7.56 ± 0.42	0.81 ± 0.10	0.25 ± 0.03	0.16 ± 0.01	1.13 ± 0.28

[1] Folic acid (100 ug) 10 min prior to ^{99m}Tc-HYNIC-D_1-FA_2.

A biodistribution study with BALB/c mice was performed to evaluate the distribution of the radiotracer (see Table S1). All animal studies were carried out in compliance with the national laws related to the conduct of animal experimentation. It was shown that the uptake intensity of kidney gradually increased and reached a maximum at 4 h (81.04% ± 4.19%ID/g, 94.78% ± 8.01%ID/g, 111.30% ± 22.25%ID/g at 1 h, 2 h, and 4 h prior to radiotracer administration, respectively). Clearance from the blood was fast and only minimal radioactivity retention in blood at 1 h point (decreased from 7.56% ± 0.42%ID/g at 5 min to 0.81% ± 0.10%ID/g at 1 h). Other organs, such as heart, liver, stomach, lung and intestine, the uptake of ^{99m}Tc-HYNIC-D_1-FA_2 kept at a low level. The kidney uptakes were competitively blocked by administration of excess folic acid 10 min prior to the radiotracer injection (12.35% ± 2.90%ID/g at 1 h after injection). These results were encouraging, because they implied the specificity of ^{99m}Tc-HYNIC-D_1-FA_2 for folate receptor. These specific properties make the radiotracer suitable for tumor detection.
